# Phage display as a tool for vaccine and immunotherapy development

**DOI:** 10.1002/btm2.10142

**Published:** 2019-09-18

**Authors:** Krystina L. Hess, Christopher M. Jewell

**Affiliations:** ^1^ U.S. Army Combat Capabilities Development Command Chemical Biological Center Aberdeen Proving Ground Maryland; ^2^ Fischell Department of Bioengineering University of Maryland College Park Maryland; ^3^ Robert E. Fischell Institute for Biomedical Devices College Park Maryland; ^4^ Department of Microbiology and Immunology University of Maryland Medical School Baltimore Maryland; ^5^ Marlene and Stewart Greenebaum Cancer Center Baltimore Maryland; ^6^ U.S. Department of Veterans Affairs Baltimore Maryland

**Keywords:** bacteriophage, biomaterials, drug delivery, immunology, nanotechnology, phage display, vaccine

## Abstract

Bacteriophages, or phages, are viruses that specifically infect bacteria and coopt the cellular machinery to create more phage proteins, eventually resulting in the release of new phage particles. Phages are heavily utilized in bioengineering for applications ranging from tissue engineering scaffolds to immune signal delivery. Of specific interest to vaccines and immunotherapies, phages have demonstrated an ability to activate both the innate and adaptive immune systems. The genome of these viral particles can be harnessed for DNA vaccination, or the surface proteins can be exploited for antigen display. More specifically, genes that encode an antigen of interest can be spliced into the phage genome, allowing antigenic proteins or peptides to be displayed by fusion to phage capsid proteins. Phages therefore present antigens to immune cells in a highly ordered and repetitive manner. This review discusses the use of phage with adjuvanting activity as antigen delivery vehicles for vaccination against infectious disease and cancer.

## Graphical abstract

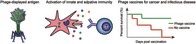

## 1 INTRODUCTION

Bacteriophages, or phages, are prokaryotic viruses that specifically infect bacteria, and are the most abundant life form on earth.^1^ Phages were first discovered in the early 20th century and noted for their antibacterial activity. The practice of administering phages (i.e., phage therapy) to patients suffering from bacterial infections began shortly after their discovery, but was controversial largely due to lack of mechanistic knowledge and variable success rates. In fact, the treatment was largely abandoned upon the discovery of antibiotics.^2^ Research in this field was reenergized, however, following the development of phage display systems in 1985.^3^ George Smith, a chemist at the University of Missouri, demonstrated fusion of peptides to the outer (i.e., capsid) proteins of phages enabling surface display. This work, for which Smith shared a Nobel Prize in 2018, laid the ground work for affinity selection, epitope mapping, and antibody discovery. Since this time, phages have been an important tool for the bioengineering field, exploited for a range of applications including theranostics,^4^ batteries,^5^, ^6^ drug delivery,^7^ and vaccine development.^1^ Phages are one of many nanotechnologies being investigated for these applications. This review will focus on the advantages that phages provide to the nanomedicine field, specifically vaccination and immunotherapy. Nanomedicine ranges from diagnostics to treatments and relies on the fields of biomedical and chemical engineering, physics, and materials science to develop new technologies. Phage display technology has been explored in vaccines against infectious disease and immunotherapies for cancer due to their intrinsic immunogenicity and ability to display foreign antigens. These advantages, as well as important immunology background, will be discussed in detail in the following section.

## 2 PHAGES ACTIVATE BOTH THE INNATE AND ADAPTIVE IMMUNE SYSTEMS

**Figure 1:**
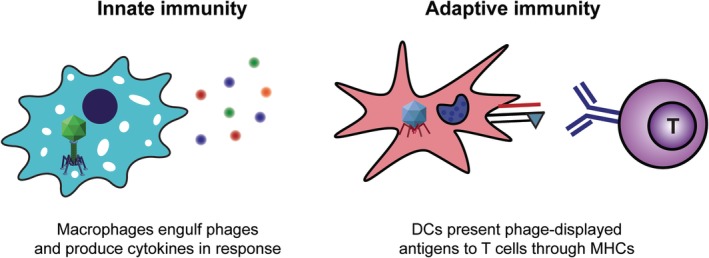
Phages interact with phagocytic and antigen‐presenting cells to activate both innate and adaptive immune responses to antigens displayed on their surface

The innate immune system is the body's first line of defense against pathogens such as bacteria and viruses. Host cells express pattern recognition receptors (PRRs) that detect danger signals known as pathogen‐associated molecular patterns (PAMPs) and damage‐associated molecular patterns (DAMPs).^8^ Examples of such signals include motifs in viruses or bacteria, or proteins released from cells due to membrane damage. One set of PRRs, toll‐like receptors (TLRs), is specifically known to recognize bacterial and viral products.^9^ For example, TLR‐4 binds lipopolysaccharide (LPS), which is present on the surface of Gram‐negative bacteria. TLR‐9, on the other hand, recognizes deoxycytidylate‐phosphate‐deoxyguanylate (CpG) regions in bacterial DNA.^10^ Interestingly, phages can interact with a variety of TLRs, which activates innate immune pathways.^11^ Phages are thought to contain CpG nucleotides within their genome and can interact with LPS following bacterial cell lysis and release. This intrinsic immunogenicity makes phages an intriguing nanotechnology candidate for vaccine carriers. When TLRs are activated, downstream signaling induces the production of inflammatory signals known as cytokines (Figure 1). These signals recruit immune cells to sites of infection or immune tissues like lymph nodes and can promote the development of an adaptive immune response. This type of response is initiated by an antigen‐presenting cell (APC) such as a dendritic cell (DC) engulfing a pathogen. A piece of the pathogen that interacts with specific cell receptors, termed an antigen, is then processed and presented on the APC cell surface through protein assemblies called major histocompatibility complexes (MHCs). Because phages are foreign to the human immune system, they can be engulfed by APCs. If the phages are also engineered to express a foreign antigen, the APC can process and present that antigen through MHCs to T cells. Naïve T cells are generated with receptors specific for an antigen and become activated toward effector or memory functions by recognition of the presented antigen along with costimulatory factors upregulated on the DC.^12^ When an antigen is presented in MHC‐I, the complex can be recognized by a CD8^+^ T cell specific for that antigen. This cell can differentiate into an effector cell known as a cytotoxic T lymphocyte (CTL) capable of directly killing cancer cells or infected host cells to stop the spread of intracellular pathogens (e.g., viruses). Phage‐based vaccines that aim to induce cell death, therefore, often display an antigen that can be presented in MHC‐I. Alternatively, when antigen is instead loaded into MHC‐II, a CD4^+^ T cell can recognize the complex and trigger the development of effector T cells known as T helper (T_H_) cells if the APC also displays costimulatory ligands. T_H_ cells assist B cells in producing high‐affinity, antigen‐specific antibodies. These secreted antibodies can bind and tag pathogens for neutralization or destruction by other immune cells. If a DC instead displays an antigen without costimulatory factors, a regulatory T (T_REG_) cell can develop. T_REGS_ maintain tolerance to “self” antigens by suppressing inflammatory cells targeted at molecules like host proteins. While tolerance normally prevents the immune system from attacking the host, its implications in cancer will be discussed below. Of particular interest to vaccines, which typically deliver an antigen and an immunostimulatory signal, is a process called cross‐presentation. In this process, an antigen that would normally be presented by MHC‐II is presented in MHC‐I, allowing a CTL response to develop. The phage‐based vaccines described in this review aim to produce antigen‐specific CTLs and/or antibodies to combat infectious disease and cancer. In addition to these effector functions, vaccines aim to induce long term protection by creating a memory response where upon antigen re‐exposure, memory B cells secrete antibodies and memory T cells rapidly polarize toward a CTL phenotype.

While not the focus of this review, it is important to note that the natural immunostimulatory nature of phages is of particular significance to their use as antibiotics. Not only does the immunogenic nature of phage particles support the development of inflammatory immune responses, the lysis of bacterial cells following phage infection can also release PAMPs capable of activating the innate immune system. Phage therapy, which has shown success in animal models through a number of routes of administration including inhalation^13^, ^14^ and dermal/transdermal,^15^, ^16^ has been widely reviewed.^17^, ^18^, ^19^, ^20^ Phages are generally considered nontoxic to humans but not all phages are suitable for therapeutics due to issues with absorption, distribution, and survival in human hosts.^21^ Despite this, the innate and adaptive immune responses induced by phages make them an attractive tool for vaccine development. A variety of nanotechnologies have been investigated for similar applications such as polymer nanoparticles encapsulating or decorated with antigens and adjuvants^22^ and self‐assembled immune polyplexes.^23^, ^24^, ^25^, ^26^ The following sections discuss the specific and unique advantages provided by nano‐sized phages beginning with structure of the vectors, with a specific focus on display of foreign antigens on the phage surface.

## 3 PHAGE GENOMES CAN BE MODIFIED TO PROMOTE DISPLAY OF FOREIGN ANTIGENS

Phages are one of the most diverse sets of organisms in existence. They consist of proteins and nucleic acids with genomes that can be comprised of DNA or RNA, which can be single stranded (ss) or double stranded (ds). While all phage genomes are encapsulated by a protein capsid, their shapes vary widely. In phage display, a foreign gene sequence encoding a peptide or protein of interest is inserted into a coat protein gene, resulting in a “fusion” protein.^27^ Through the inherent phage machinery, this protein is subsequently displayed on the surface of the phage where it retains its affinity and specificity for the target receptor. Phages have been used for a variety of applications including gene delivery,^28^ antibody identification,^29^ tissue engineering scaffolds,^30^ and biosensors,^31^, ^32^, ^33^ but the main focus of this review is application of phage display vaccine and immunotherapy development. Families of phages vary greatly in many ways including size, shape, and surface proteins used most frequently for antigen display. To provide context for the subsequent sections on phages as vaccine tools, this section will discuss the structure and biology of six families of phage.

### 3.1 Filamentous phage

Filamentous phages of the *Inoviridae* family are typically about 900 nm long and only 7 nm wide (Table 1).^34^ Here, we discuss strains of the Ff group including M13 and fd. The major coat protein pVIII, which creates the side wall that shields the circular 6.4 kb ssDNA genome, is present in 2,700 copies. Additionally, there are two minor coat proteins, pIII and pVI, at one end of the phage and two other minor coat proteins, pVII and pIX, at the other end. This family of phages specifically infect *Escherichia coli* (*E. coli*) that have an F pilus. The N‐terminal of pIII first attaches to the F pilus, allowing the viral DNA to enter the cytoplasm. As the cellular machinery produces progeny genomes, they exit through the cell envelope where they acquire coat proteins. Importantly, filamentous phage can be continuously created and secreted by the host bacterium without killing it. This provides an opportunity to produce phages in a rapid and sustained manner, which could be beneficial for quickly developing vaccines against emerging threats. Another advantage of the filamentous phage display system is that all five coat proteins can display antigens. It is considered one of the most efficient display systems and is widely used in the field. pVIII is most often used due to its high copy number, but it can only display short peptides since it must be extruded through the cell envelope.^35^ pIII by contrast can display large proteins but at a much lower density, which reduces immunogenicity.

**Table 1:** Structure and application of families of phages used as vaccine platforms

Phage	Filamentous	T4	T7	λ	MS2	Qβ
**Structure**	Filamentous 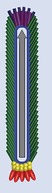	Icosahedron + tail 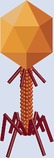	Icosahedron + tail 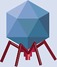	Icosahedron + tail 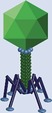	Icosahedron 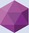	Icosahedron 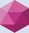
**Major dimensions**	900 nm × 7 nm	120 nm × 86 nm	56 nm × 29 nm	60 nm × 150 nm	26 nm	28 nm
**Vaccine applications**	Parasites Fungi Influenza Melanoma Breast cancer	Anthrax Plague Lung cancer	Influenza Foot and mouth disease Epstein–Barr Melanoma Breast cancer	Hepatitis B Breast cancer Liver cancer	Foot and mouth disease HPV Zika	Breast cancer Melanoma

### 3.2 T4 phage

T4 is a lytic phage strain of the *Myoviridae* family that features an elongated icosahedral head that is approximately 120 nm by 86 nm, a tail, and tail fibers (Table 1).^12^ The capsid proteins of the phage are defined as the major capsid protein gp23, the vertex protein gp24, the portal protein gp20, the small outer capsid protein (Soc), and the highly antigenic capsid protein (Hoc). The T4 phage genome is 171 kb dsDNA that exits through the portal vertex formed by gp20 to infect bacteria. Upon infection, the phage enters a vegetative state and must lyse the cell to release viral progenies.^36^ Soc forms a cage around the head of the phage to stabilize it against extreme heat and pH as well as enzymatic degradation, which is useful not only for vaccine administration inside the human body but also for transport to developing nations.^37^ Both the Soc and Hoc proteins have been reported for antigen display, with the N‐ and C‐termini of the 870 copies of Soc both available to display peptides without affecting recognition by immune cells. By contrast, the C‐terminus of the 155 copies of Hoc has the binding site, making the N‐terminus more desirable for peptide display.^38^ It has also been demonstrated that the Hoc protein can facilitate binding to mammalian cells, potentially enabling enhanced vaccine delivery characteristics.

### 3.3 T7 phage

T7 is also a lytic phage, but from the *Podoviridae* family, with a 56 nm icosahedral head composed of two major capsid proteins, gp10A and gp10B, and containing a 40 kb dsDNA genome (Table 1).^39^ Other major proteins constituting the phage particle include the connector protein gp8, the tail proteins gp11 and gp12, and the tail fiber protein gp12.^3^ The 29 nm tail facilitates binding to bacteria and the proteins gp15 and gp16 assist in DNA insertion. Like T4, the host cell is lysed to release new phage particles. Since gp10B is not essential for assembly of the capsid, it has been explored for antigen display at the C‐terminus with small peptides in high copy numbers, or larger proteins with low to mid copy numbers. Interestingly, the density of antigens displayed on particulate materials has been shown to influence the type of immune response that develops, which implies a delicate balance between using phages to display antigens in low or high copy numbers.^40^

### 3.4 λ phage

λ phage, a strain of the *Siphoviridae*, features a 60 nm icosahedral head with an approximately 4 nm shell thickness and a flexible tail about 150 nm long (Table 1).^36^ The phage consists of the major capsid proteins gpD and gpE, the portal protein gpB, the scaffolding protein gpNu3, the viral protease gpC, and the major tail protein pV. Interestingly, λ is a temperate phage that, upon cell infection, can enter a vegetative state of growth or a lysogeny state where the genome is stored in the cell. The large 48.5 kb linear dsDNA genome is commonly employed for DNA vaccination strategies. In addition, the gpD protein is ideal for antigen display as there are up to 420 copies on the phage and both the N‐ and C‐termini are available for fusion.^41^, ^42^ Additionally, the C‐terminus of the pV tail protein can be utilized for display, providing an opportunity for fusion of multiple antigens per phage particle.

### 3.5 MS2 phage

MS2 phage is part of the *Leviviridae* family which are the smallest and simplest phages (Table 1). Size of antigen‐delivering particles can impact trafficking to infection sites and immune tissues and the subsequent response that develops.^43^ This small family of phages features an icosahedral size of 26 nm, which my facilitate rapid draining to lymph nodes where displayed antigens can be displayed to immune cells. The 3.57 kb ssRNA genome encodes the major coat protein CP, the maturation protein A, the replicase, and the lysis protein L.^44^ MS2, like filamentous phage, binds the F pilus of *E. coli* to insert its genome.^34^ Unlike filamentous phage, however, the release of new viral particles requires lysis of the host cell. While both the C and N‐termini are essential for assembly of the 180 copies of CP, there is an exposed β‐hairpin that has been exploited for antigen display.

### 3.6 Qβ phage

Qβ phage belongs to the same family as MS2 phage, meaning it also has a short (4.2 kb) ssRNA genome that only includes a few proteins: the major coat protein CP, the maturation protein A2, and the replicase (Table 1).^45^ The 28 nm icosahedral phage also binds the F pilus to infect host cells where A2 inhibits cell wall synthesis causing lysis and the release of new Qβ phage particles.^46^ The A1 protein is occasionally produced due to a leaky stop codon and contains a 196‐amino acid extension of the C‐terminus of CP. The read‐through domain of A1 is essential for host cell infection and is the site most often used for antigen display.^47^ The following sections outline several examples of phage display being used in infection and cancer. We begin by describing phage‐based vaccines that display antigens associated with parasites, fungi, viruses, and bioterrorism agents.

## 4 PHAGES AS A VACCINATION PLATFORM AGAINST INFECTIOUS DISEASES

Vaccines are typically used as prophylactics against viruses or bacteria. Inactivated vaccines are created by killing a pathogen and often require multiple doses to be effective.^1^ Live attenuated vaccines, on the other hand, are created from a live pathogen that has been altered so that it is no longer infectious. Because live attenuated vaccines are similar to the original virus or bacteria, they often require smaller doses to generate robust immune responses. Subunit vaccines contain only a piece of the pathogen such as a protein or polysaccharide that has proven to be antigenic, while nucleic acid vaccines deliver bioengineered DNA or RNA to host cells for incorporation into the genome and subsequent production of antigens. These conventional vaccine types have greatly reduced the rates of many transmissible diseases and have even resulted in the eradication of smallpox.^48^ Despite the successes in this field, challenges still remain in the synthesis of vaccines against many persistent and emerging threats. An important aspect of vaccine development is selection of the appropriate antigen and adjuvant. Vaccine components that can stimulate inflammatory signaling through TLR pathways, such as CpG motifs in the phage genome, are of particular interest as adjuvants due to their ability to generate strong, antigen‐specific immune responses. It should be noted, however, that TLR‐based adjuvant administration in humans has resulted in side effects ranging from injection site pain to renal and hepatic impairment.^49^ One approach to phage‐based vaccination is to display a foreign antigen as a fusion protein on the capsid surface.^50^ Another approach involves the direct conjugation of an antigen to the surface of the phage without altering the genome. While the genome of phages can be engineered to synthesize nucleic acid vaccines, this review focuses on the display of foreign antigen on the phage surface. Phages are also attractive targets for vaccination vehicles because studies have demonstrated that surface displayed antigens can be loaded into either MHC‐I or MHC‐II which can result in both a CTL and an antibody‐mediated response. This is accomplished because a phage particle is recognized as foreign and can be taken up by APCs.^51^ Additionally, because phages only infect prokaryotes they are generally considered safe for use in humans.^52^ Many of these described advantages of phage‐based vaccines are similar to other nanotechnology platforms. For example, synthetic nanoparticles are also phagocytosed by APCs and can be decorated with or used to encapsulate antigens and/or adjuvants. One important difference, however, is that the cost‐effective production of uniform nanomaterials on a large scale is still a challenge. Phages offer the advantage of rapid and uniform replication, allowing for inexpensive and sustainable production on a large scale. Phages have been researched for vaccination against foot and mouth disease,^53^ hepatitis B,^54^ Epstein–Barr virus,^55^ and numerous other infectious diseases. Some examples are highlighted in detail below.

### 4.1 Filamentous phage

As mentioned, one advantage of phages is the ability to interact with TLRs to produce robust immune responses. One group aimed to characterize the dependence of phage‐induced immunity on TLR9 signaling. The ovalbumin (OVA) protein from chicken eggs is often used as a model antigen in vaccines studies because it is foreign to mice and humans and is immunogenic. In this study, filamentous fd phages were engineered to express either a peptide of OVA or peptides from the parasite *Trypanosoma cruzi* on the pVIII major coat protein. When B6 mice immunized with OVA‐phage were challenged with OVA‐expressing *T. cruzi*, the blood and myocardium parasitemia levels were significantly lower than that of control mice. In contrast, in transgenic mice where TLR9 is knocked out, OVA‐phage immunization was unable to protect the mice from parasite challenge. Interestingly, phages were capable of inducing maturation of bone marrow dendritic cells (BMDCs) isolated from B6 mice, but not from TLR9^−/−^ mice. Taken together, these results suggest that the induction of an antigen‐specific immune response capable of protecting mice from infection is dependent on TLR9 signaling. Importantly though, phages may have the capability of interacting with a wide range of TLRs, so further studies are needed to characterize the importance of each individual receptor and subsequent downstream signaling in generating immunity.

Certain fungi and viruses, including *Candida albicans* (CA) and influenza, infect human hosts through the mucosal route (i.e., mouth, nasal passages).^56^, ^57^ For this reason, there is increasing interest in administration of vaccines through the mucosal route to generate efficient, lasting protection at the sites of infection.^58^ In the case of oral administration, the vaccine first reaches the gut where it interacts with the gut‐associated lymphoid tissue (GALT), a collection of immune tissues that is partially independent of the other secondary immune tissues. This complex system involves a number of immune tissues including aggregated lymphoid follicles located in the small intestine, known as Peyer's patches, and mesenteric lymph nodes.^59^ The GALT samples circulating antigens mainly through microfold (M) cells, which are capable of phagocytosing bacteria and other materials. For example, the gram‐negative bacteria Yersinia pseudotuberculosis interacts with M cells through expression of a surface adhesion protein called invasin which binds β1 integrins on the M cell surface. Harnessing this interaction, one group bioengineered *E. coli* cells to express invasin.^56^ Next, filamentous fd phage were engineered to express amino acids 2–16 of the Matrix protein 2 of influenza A (M2e) on pVIII. M2e has been shown to generate strong antigen‐specific responses in animal models and is highly conserved in a virus known for rapid mutation. The invasin‐expressing bacteria were then infected with the M2e phage and delivered orally to mice, where it was confirmed they accumulated in Peyer's patches. Additionally, after six administrations of the phage‐infected cells, M2e‐specific IgG antibodies were found in mice. This resulted in protection from a sublethal does of mouse‐adapted influenza A as evidenced by significantly reduced weight loss and lung virus titers compared to control mice. It should be noted, however, that the IgG titers were relatively low and that an IgA response to M2e was not found in treated mice which is normally a hallmark of successful mucosal vaccination. Additionally, there are notable regulatory and compliance challenges with a vaccine strategy that incorporates live bacteria, leading the authors to suggest this platform may be more applicable to immunization of livestock animals.

**Figure 2:**
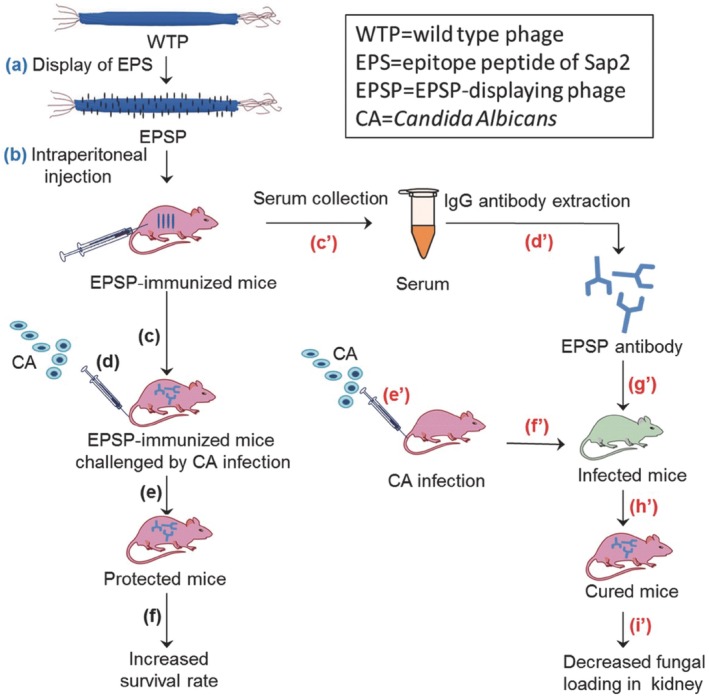
Filamentous phages displaying the EPS peptide of Sap2 were injected into mice that were then challenged with CA infection. This resulted in a decreased fungal load in the kidneys and increased survival to lethal challenge. Reprinted with permission from Huai Y, Dong S, Zhu T, Li X, Cao B, Gao X, Yang M, Wang L, Mao C. Genetically engineered virus nanofibers as an efficient vaccine for preventing fungal infection. Adv Healthcare Mater. 2016;5(7):786–794^60^

In another study aimed at infections of the mucosal tract, filamentous phage was used as an immunization strategy against CA.^60^ This fungus is especially dangerous to patients with comprised immune systems, where it has a high morbidity and mortality rate.^61^ Secretory aspartyl proteinases (Saps) are considered the major antigenic determinant in CA infection, with Sap2 being the most prominent to cause virulence. In this study, a short peptide of Sap2, EPS, was chosen for inclusion in the vaccine because it was shown to be recognized by IgG antibodies from patients with CA infections. The many copies of the pVIII protein were also harnessed by this group to display EPS through fusion along the side wall of the filamentous phage (Figure 2). In mice, immunization with EPS‐expressing phage resulted in T_H_1 and T_H_17‐like responses as seen by the secretion of cytokines such as IL‐2, IL‐12, IL‐17, and IFN‐γ from splenocytes. Following a CA challenge injected i.v., immunized mice displayed decreased numbers of colony forming units and lesions in the kidneys, a protection not offered by immunization with Sap2 alone. Despite this, the survival rate to a lethal challenge was 43.75% for ESP‐displaying phage and 56.25% for Sap2 immunized mice. As mentioned above, CA infection is associated with the mucosal tract. As there is some evidence to suggest that immunization via the mucosal route can enhance protection against viruses and fungi that act here, it would be interesting to see this strategy tested via oral administration rather than i.p. injection.

### 4.2 T4 phage

An application of immunization that has not yet been discussed is vaccine development for bioagent threats. Anthrax, caused by the bacteria *Bacillus anthracis*, is extremely dangerous due the ease of spreading infectious spores.^62^, ^63^, ^64^ In 1979 for example, *B. anthracis* was inadvertently released from a bioagent production facility in Sverdlovsk, Russia and killed 68 people. In the United States, *B. anthracis* was weaponized by mailing spores to political and news media offices. This resulted in exposure to 65 people, infection of 22 people, and death of five people. Virulence is partially due to the tripartite anthrax toxin which includes lethal factor (LF), edema factor, and protective antigen (PA). PA is required for the formation of the other two components, and is therefore considered an attractive target in vaccination strategies. One group employed the T4 phage system to display PA and protect rabbits from anthrax.^65^ When a range of commonly used adjuvants were administered along with PA to rabbits, all achieved 100% protection from a lethal pulmonary challenge with *B. anthracis* Ames strain spores. When PA was instead displayed on the Hoc and Soc proteins of the T4 phage head, only 80% of rabbits were protected from lethal challenge. In nonhuman primates however, this vaccination strategy resulted in 100% protection.^66^ This difference underlines the challenges associated with clinical translation of vaccines. Phage‐based vaccines have the unique advantage of facile and rapid modification. This implies that as a vaccine is tested in various animal models, it can quickly be adjusted through altering the phage genome in cases where efficacy is in question.

**Figure 3:**
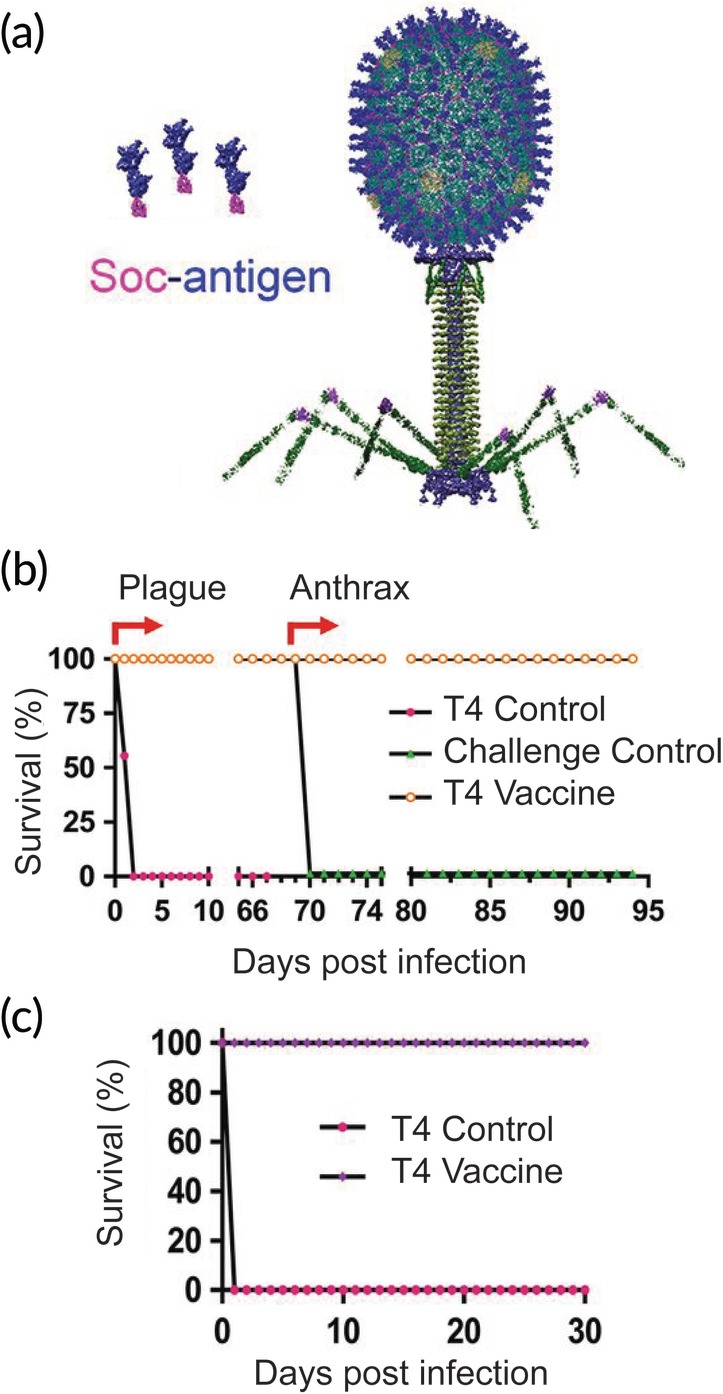
T4 phages expressing antigens from plague and anthrax protect rats from challenge. (a) PA from *B. anthracis* and F1 and V from *Y. pestis* were fused to the Soc protein on the T4 phage head. (b) When immunized rats were challenged with *Y. pestis*, 100% survived. LF and PA challenge of these same rats also resulted in 100% protection. (c) In a simultaneous challenge model, 100% of immunized rats survived. Adapted with permission^67^

In another study, the T4 phage display platform was exploited to induce protective immunity against both anthrax and plague.^67^ Three plague pandemics have been documented in human history, and the bacteria has also been employed as a biological warfare agent.^62^, ^68^ The “Black Death” resulted in the elimination of up to one third of Europe's population, and parts of the United States are still exposed to the plague‐causing bacteria *Yersinia pestis* each year.^69^ Here, PA and two antigens from *Y. pestis* (F1 and V) were fused to Soc (Figure 3a). Immunization success was tested in a sequential challenge model where animals were first injected i.p. with PA and LF from *B. anthracis*, followed 33 days later by *Y. pestis*. Although only 80% of mice survived the first challenge, all of these surviving mice were protected from the second challenge. When the two challenges were instead administered simultaneously, 88% of mice survived. When these two models were employed in rats, however, 100% survival rates were witnessed for all challenges (Figure 3b,c). These impressive results were found to be the result of strong T_H_1 and T_H_2 responses that resulted in IgG1 and IgG2a antibodies against the antigens from both bacteria types. This work demonstrated the potential for exploiting the multivalency of T4 phage to create vaccines that protect against multiple infectious agents. Due to the ease and speed with which T4 phage displayed antigens can be modified, it presents a potential platform against other bioagent threats.

### 4.3 T7 phage

Filamentous phage displaying M2e was described above as an influenza A vaccine. Another group used a similar strategy to instead display M2e on the surface of lytic T7 phage.^70^ This is a highly researched field because of the annual public health burden and the desire to create a “universal” vaccine. In fact, approximately 40 million people in the United States developed the flu during the 2018–2019 season which results in an annual $11 billion burden to the economy.^71^, ^72^ Current vaccines aim to generate antibodies against hemagglutinin which is actually subject to antigenic drift where mutations occur specifically in antibody‐binding sites, resulting in a need to manufacture new vaccines for flu season each year. M2e, by comparison, is highly conserved, resulting is a large focus of research efforts with some even reaching phase 1 clinical trials.^73^, ^74^ Despite some success, M2e on its own is poorly immunogenic, likely because it is not highly expressed. One reason that T7 phage could overcome this challenge is its multivalent nature that allows repeated, organized display of antigens, a key determinant of immunogenicity to B cells. When mice were immunized with M2e fused to the gp10B capsid protein of T7 phage, high levels of antigen‐specific IgG1 and IgG2a antibodies were induced. Although phage has some adjuvanting characteristics, the inclusion of the strong Complete Freund's Adjuvant (CFA) significantly elevated antibody levels. Of note, mice that had been immunized with M2e‐phage and CFA were 100% protected from lethal challenge with both the H1N1 and H3N2 strains of influenza, demonstrating cross‐protectivity. By comparison, mice that received only M2e‐phage had survival rates of 66% and 83%, respectively. While these results are promising, CFA is not currently approved for use in humans. The adjuvanticity of phages therefore needs to be improved without the inclusion of a strong antigen, which may be possible through modification of different surface proteins.

### 4.4 λ phage

An important consideration when developing a new platform for vaccination is how it improves beyond the current state of the art. For example, there is a commercially available vaccine for hepatitis B that is recommended to be included in the vaccine schedules for all children by the World Health Organization.^75^ This can be a significant problem in developing countries because the three‐dose immunization is more expensive than every other recommended vaccination combined. Despite this burden, it is an extremely important vaccination as hepatitis B is highly contagious and leads to approximately 780,000 deaths each year.^76^ Development of phage vaccines represents a unique opportunity to rapidly and cheaply produce large quantities. One group compared the efficacy of the currently used protein vaccine Engerix B to λ phages displaying the small surface antigen of hepatitis B (HBsAg).^77^ Interestingly, phage immunization resulted in an increased antibody response at multiple timepoints, including both IgG and IgM antibodies. Additionally, when lymphocytes isolated from immunized mice were restimulated with HBsAg, high levels of proliferation were seen for both vaccination groups. To obtain clinically relevant results, it will be important to follow up on this work to compare how mice immunized with either vaccine are protected from a hepatitis B challenge model.

### 4.5 MS2 phage

In another study comparing phage immunization to a commercially available vaccine, one group displayed a conserved epitope from the minor capsid protein L2 of human papillomavirus (HPV) to Gardasil‐9.^78^ There are approximately 40 different types of HPV that are sexually transmitted and can cause the development of genital warts and cancer. For this reason, vaccines need to be extremely broad. The three currently licensed vaccines, however, are all mainly derived from the major capsid protein L1, which offers little cross‐protection because it is not highly conserved across the many types of HPV. This has led researchers to focus on the very highly conserved L2 protein for vaccine synthesis. While L1 can form virus‐like particles on its own, L2 cannot, leading these researchers to display the epitopes of interest on the surface of MS2 phage. MS2 phages were developed displaying peptides from: epitopes of L2 of HPV31 and L2 of HPV16, a consensus L2 peptide from 23 types of HPV, or both peptides. Regardless of the L2 peptide(s) displayed, a robust antibody response was observed in immunized mice. Interestingly, when the phage particles were mixed together, the response was neither enhanced nor diminished. Next, immunized mice were challenged by vaginal infection with HPV16, 31, 45, and 58. In this case, the group immunized with a mixture of L2 peptide‐displaying phages were the most protected from challenge in three of the four HPV types. While the phages did not significantly outperform Gardasil‐9 in any of the challenge models, the technology has many other advantages including ease and cost of manufacturing. Additionally, the ability to engineer phages to display multiple different antigens in high valency encourages additional studies into cross‐protectivity. Many of the goals for inducing a robust protective response against infectious agents are similar to what is needed to generate antitumor immunity. In the next section, examples of phage display for cancer vaccination and treatment are discussed.

## 5 PHAGE AS A TUMOR ANTIGEN DISPLAY TOOL

While cancer treatment and diagnosis have significantly improved in recent years, many challenges still exist. For instance, two standard treatment options, chemotherapy and radiation, have significant side effects such as nausea, hair loss, anemia, and kidney problems.^79^ Cancer immunotherapies aim to be more specific and cause less systemic toxicity by delivering tumor antigens and strong adjuvants that may generate a robust antigen‐specific immune response. One significant challenge is the tumor microenvironment, which contains a high frequency of T_REGS_ that suppress inflammatory immune cells in part through upregulation of checkpoint molecules.^80^ Immune checkpoints mainly function to halt inflammatory responses to “self” molecules but are overexpressed in certain cancers. The intrinsic immunogenicity of phages may present an opportunity to overcome this suppressive, or tolerogenic, environment, while phage display can be exploited to deliver tumor antigens to generate a more specific response. As discussed in the previous section, phages are capable of inducing antigen presentation in both MHC‐I and MHC‐II through a process known as cross presentation. This feature is also extremely advantageous when developing cancer immunotherapies as CTLs activated by MHC‐I recognition are thought to be crucial to killing tumor cells. Additionally, there is increasing evidence that the humoral response that could be induced by antigen recognition in MHC‐II can play a role in tumor destruction.^81^ Another important feature of phages is activation of the innate immune system through interaction with PRRs which causes the release of inflammatory cytokines capable of altering the immunosuppressive environment that surrounds a tumor. The combinatorial approach offered by phages to be phagocytosed by APCs and efficiently deliver a high density of tumor antigens while also stimulating immune signaling offers great promise. As mentioned earlier, ligand density influences the type of immune response that develops and is of particular importance to response to cancer therapeutics.^82^ Phage display vaccines have been targeted toward cancers such as breast,^83^, ^84^ liver,^85^ and lung,^86^ and some examples are discussed below in detail.

### 5.1 Filamentous phage

**Figure 4:**
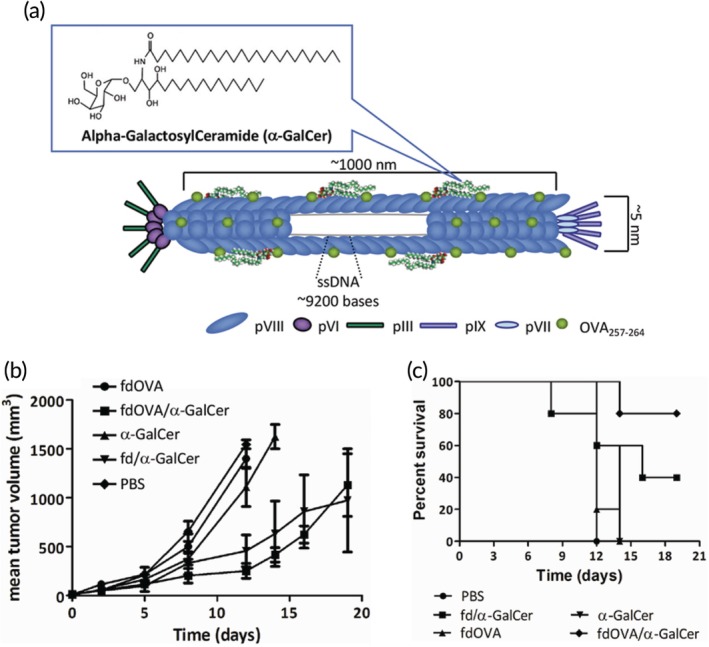
Phages delivering an immunostimulatory (glyco)lipid convey antitumor effects. (a) alpha‐GalactosylCeramide was conjugated to the pVIII coat protein of fd phage. Therapeutic treatment in a mouse melanoma model resulted in delayed tumor growth (b) and increased survival (c). Adapted with permission^87^

While much of cancer research is focused on the generation of CTLs with tumor cell killing capabilities, invariant natural killer T (iNKT) cells have also demonstrated an antitumor potential.^87^ iNKT cells appear to be involved in both innate and adaptive immunity and are activated by recognition of (glyco)lipid antigens presented by APCs. One such antigen, alpha‐GalactosylCeramide (α‐GalCer), induces the secretion of a variety of inflammatory cytokines which in turn can activate T cells, B cells, and DCs.^88^ Due to the strong stimulatory properties of this antigen, it can induce exhaustion in iNKT cells as evidenced by increased expression of checkpoint molecules. As discussed, inclusion of a strong adjuvant is crucial to overcoming tolerogenic barriers such as unresponsiveness, or anergy, of T cells. One group used filamentous fd phage for this purpose, conjugating α‐GalCer to the surface through lipid binding to the hydrophobic regions of the pVIII protein (Figure 4a).^87^ The phages demonstrated an ability to be phagocytosed by DCs in vitro where they presented α‐GalCer to iNKT cells. While cells isolated from mice that had been immunized with α‐GalCer did not respond to restimulation, suggesting anergy, splenocytes from mice that received phage‐conjugated α‐GalCer were still responsive. To test the platform as a cancer immunotherapeutic, the antigenic determinant of OVA was displayed as a fusion protein on pVIII. Immunization in mice resulted in an antigen‐specific T cell response that resulted in IFN‐γ production. In the B16‐OVA melanoma model, mice were treated by intratumoral injection, which delayed further tumor growth (Figure 4b) and increased survival (Figure 4c). This was likely, at least in part, due to an increased frequency of CD8^+^ T cells that infiltrated the tumors of mice that were vaccinated with α‐GalCer and OVA displayed on phages. This strategy represents a unique use of phages to codeliver antigen and adjuvant through both phage display and exploitation of hydrophobic residues for direct conjugation. As lipids are gaining increasing attention in immunotherapeutics for their stimulatory properties, phage is an important delivery tool to consider. Additionally, there is increased clinical interest in the intratumoral injection route for cancer vaccines, especially in patients who develop inadequate CTL responses.^89^

**Figure 5:**
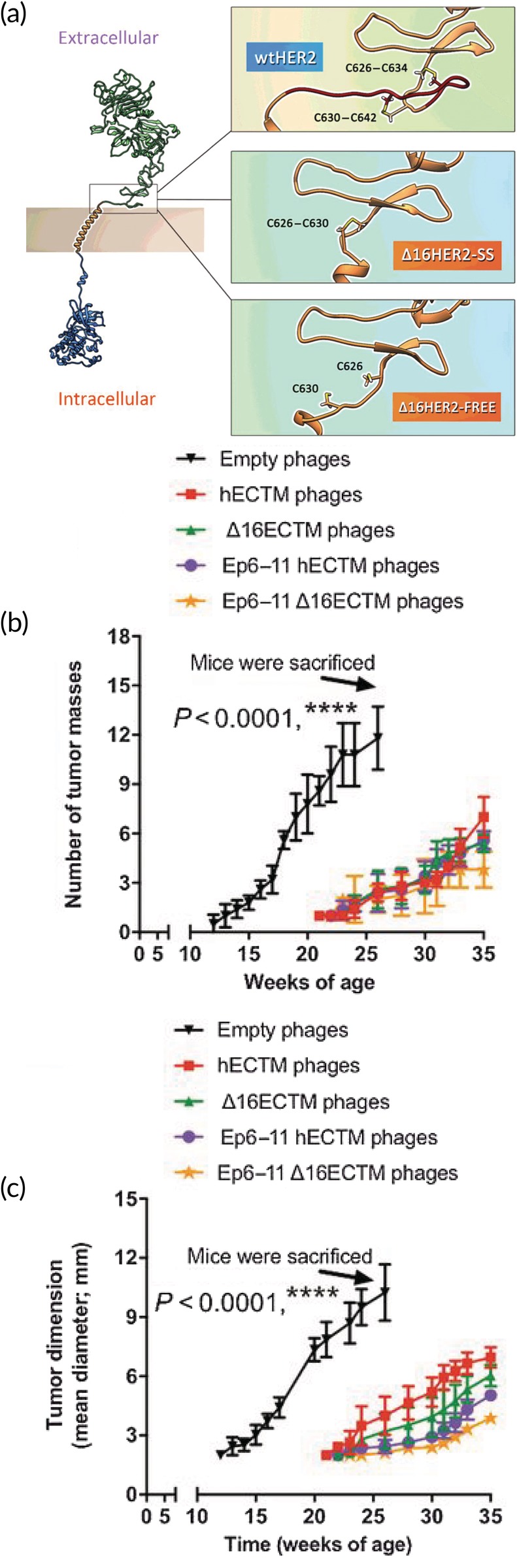
Vaccination of transgenic mice that spontaneously develop breast cancer with a phage‐displayed HER2 variant (a) thought to be associated with oncogenesis results in decreased tumor frequency (b) and volume (c). Reprinted by permission from the American Association for Cancer Research: Bartolacci C, Andreani C, Curcio C, et al., Phage‐based anti‐HER2 vaccination can circumvent immune tolerance against breast cancer. Cancer Immunol Res. December 2018;6(12):1486–1498. doi:10.1158/2326‐6,066.CIR‐18‐0179^97^

Breast cancer is classified into five different stages based on tumor size, invasiveness, and metastasis.^90^ If patients are first diagnosed with later stages, the 5 year survival rate can be as low as 27%.^91^ Success in treating breast cancers is often reliant on the expression of certain receptors. For example, the tyrosine kinase receptor HER2 is thought to be overexpressed in up to 30% of patients and is correlated with more aggressive cancer and subsequently increased mortality.^92^ There are currently a number of FDA‐approved monoclonal antibody treatments aimed at HER2, but they often need to be combined with chemotherapy which comprises the patient's immune system and is generally toxic to all cell types.^93^ Immunotherapy aimed at HER2 could provide an opportunity to create a robust antigen‐specific immune response capable of destroying the tumor without being toxic to other tissues. Recent studies have revealed that a variant of HER2 that lacks exon‐16, Δ16HER2, is responsible for creating homodimers that invoke signaling pathways that ultimately lead to uncontrolled cell division (Figure 5a).^94^ Because this variant is found in a majority of patients with HER2‐positive breast cancer, it is an attractive target for immunotherapy.^95^ In fact, it is thought that resistance to monoclonal antibody treatments may be due to inability to bind to this variant form of HER2.^96^ One group created a DNA vaccination that encoded the extracellular and transmembrane domains of Δ16HER2 and demonstrated that following prophylactic immunization, mice were 100% protected from challenge with Δ16HER2‐expressing tumor cells for up to 100 days.^97^ The group next employed a model where transgenic mice express human Δ16HER2 and spontaneously develop mammary carcinomas.^98^ Unfortunately in this case, 100% of immunized mice developed tumors. A number of studies were then carried out to determine the mechanism of tolerance that prevented protective immunity. For example, it was determined that immunization failed to produce HER2‐specific antibodies but increased the frequency of T_REGS_ in the spleen. Additionally, Δ16HER2 mRNA was found in the thymus of Δ16HER2‐expressing transgenic pups, suggesting central tolerance plays a role and may result in the variant being recognized as a self‐antigen. To promote a stronger inflammatory response capable of breaking tolerance, the researchers turned to the M13 phage display system. Filamentous phage is able to cross through blood vessels, making it especially attractive for cancer applications. Δ16HER2‐expressing transgenic mice were immunized with phages displaying the extracellular and transmembrane domains of Δ16HER2 on the pIII protein. This resulted in decreased number of tumors (Figure 5b) with smaller average size (Figure 5c) and prolonged the tumor latency period. Further analysis of the mice indicated this may have been the result of an increased number of tumor‐infiltrating CD8^+^ T cells and an enhanced antibody‐dependent cell‐mediated cytotoxic response. This work highlights the importance of antigen presentation and the inclusion of a strong adjuvant when attempting to overcome the severely suppressive tumor microenvironment.

### 5.2 T7 phage

Another group targeted the overexpressed HER2 receptor by displaying an immunodominant epitope on T7 phage.^99^ There is some evidence to suggest that inclusion of a whole protein tumor antigen in a cancer immunotherapy could actually stimulate tumor cell growth. For this reason, peptide vaccines are of interest, but as discussed earlier, peptides alone are poorly immunogenic and require the inclusion of a strong adjuvant to generate an antigen‐specific response. T7 phage is a highly efficient platform for peptide display that is highly immunogenic and promotes uptake by APCs. An MHC‐I CTL‐associated epitope, p66, was displayed as a fusion protein on the gp10B capsid protein. Splenocytes isolated from phage‐immunized mice responded to restimulation with p66 by producing a significant IFN‐γ response. Notably, the cytokine production levels were higher than in cells isolated from mice immunized with p66 emulsified in the strong adjuvant CFA. In a prophylactic vaccination model, five of six mice that were immunized with p66‐phage remained tumor free following challenge with HER2‐overexpressing tumor cells. In a therapeutic model where mice were not treated until palpable tumors had formed, four of six mice cleared the tumors and did not develop new tumors for up to 80 days. This work demonstrates that the repetitive display of immunogenic peptides by the T7 phage system makes it a promising technology for overcoming immune tolerance to produce a robust anticancer immune response.

### 5.3 Qβ phage

**Figure 6:**
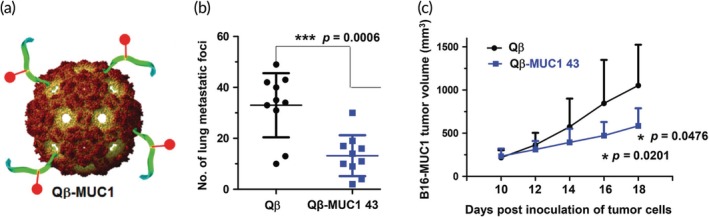
A MUC1 epitope displayed on Qβ phage reduces tumor load in mice. (a) A peptide of MUC1 selected through phage‐mediated epitope discovery was conjugated to the surface of Qβ phage and injected into MUC1 transgenic mice. (b) When the mice were also challenged with B16‐MUC1 cells, lung metastases were significantly reduced in immunized mice. (c) Qβ‐MUC1 administered along with a checkpoint blockade resulted in reduced growth of solid tumors. Adapted with permission from Wu X, Yin Z, McKay C, et al. Protective epitope discovery and design of MUC1‐based vaccine for effective tumor protections in immunotolerant mice. J Am Chem Soc. 2018;140(48):16596–16609. Copyright 2018 American Chemical Society^100^

One of the key advantages of phage that has not yet been discussed for its application to cancer immunotherapy is epitope discovery. One group exploited the Qβ phage display system to rapidly and efficiently generate a library of 20 peptides of mucin‐1 (MUC1), a glycoprotein expressed on cancer cells.^100^ MUC1 is expressed by many types of cancer cells and is therefore an interesting target for cancer immunotherapies.^101^, ^102^ By incubating phages expressing the various epitopes with sera from immunized mice, a region of interest (SAPDTRPAP) was discovered. Mice were vaccinated with this epitope conjugated to the phage surface through a flexible alkyl amide linker (Figure 6a), which generated antibodies capable of selectively binding both MUC1‐expressing B16 melanoma cells and MCF‐7 breast cancer cells. In a model of metastatic cancer, transgenic mice that constitutively express MUC1 were also injected with B16‐MUC1 cells. Phage immunization resulted in a reduction in the number of metastases in the lung (Figure 6b). Combination therapy was also explored by immunization with phage and treatment with checkpoint blockade therapy which resulted in reduced tumor volume in a B16‐MUC1 solid tumor model (Figure 6c). As described here, phage technology can be exploited to both select antigens for inclusion in vaccines and to deliver the vaccines themselves to produce a robust response against tumors.

## 6 CONCLUSIONS

Phages offer many unique features useful when aiming to develop a robust inflammatory immune response targeted at viral infection or cancer. Phage‐based vaccines, like other nanotechnologies, aim to present antigen to the immune system while simultaneously activating stimulatory pathways. Nanotechnology is researched for applications beyond vaccinology in the medical field including disease characterization, targeted drug delivery, and tissue regeneration. Phages specifically have been used in a wide array of applications ranging from biosensors to cancer immunotherapies. Reasons for this diversity include the ability to produce a large number of viral particles in a rapid and cost‐effective manner, advantageous sizes and aspect ratios, the ability to display proteins and peptides in highly‐ordered arrays, and phage safety profile in humans. In this review, we focused on phages as immunogenic carriers of antigens related to infectious diseases and cancer. The foreign nature of phages activates inflammation through innate immune pathways, while the particulate shapes promote uptake by APCs. Interaction with APCs in turn activates the adaptive immune system which can produce CTLs capable of killing virally infected or cancerous cells and antibodies capable of binding pathogens. The combination of both the innate and adaptive immune pathways is crucial in generating robust and long‐lasting antigen‐specific responses. The highlighted examples demonstrated how phage‐based activation of inflammatory pathways can prevent viral propagation, provide protection from biothreats, and clear established tumors in preclinical models. One understudied area of phage display is the design of therapeutic vaccines for autoimmune disease. A number of studies have revealed that phages can be immunomodulatory such as by decreasing reactive oxygen species production^103^ and reducing inflammatory cytokine levels.^104^, ^105^ These observations are not unique to bacteriophages as other immune signal delivery platforms such a gold nanoparticles have been shown to increase or decrease inflammation depending on parameters such as shape, surface charge, and disease model.^106^, ^107^ There are still questions about mechanism of action on the molecular, cellular, and tissue levels that need to be answered as researchers look to transition phage nanotechnology from the benchtop to clinical trials. For example, the innate response to phages in mammals has yet to be fully characterized. Although no in‐depth studies have compared different families of phage for efficacy in vaccines and immunotherapies, selection of phage display platform is a crucial step in rational design. As outlined previously, families of phages have specific properties that can be exploited for antigen delivery. For example, the ultrasmall size of phages from the *Leviviridae* family (i.e., MS2, Qβ) could promote rapid drainage to lymph nodes, where immune responses are mounted. Beyond just size, the density of antigen displayed by a delivery system is important in the development of inflammatory or tolerogenic responses.^40^ This is of particular importance to phage display where antigens can be fused to proteins that are present in low, medium, or high copy numbers based on the family of phage selected. Further, phage genomes vary widely including ssDNA, dsDNA, and ssRNA which have varying levels of potential for genetic engineering. Clinical translation of phage‐based vaccines will require analysis of each of these factors to determine the proper system for a given disease. One crucial step toward translation will be demonstrating safety in humans, which could be accelerated by the anticipated increased use of phage therapy in patients with antibiotic‐resistant bacterial infections. Despite these questions and challenges, the use of phages to induce protective and therapeutic immunity shows great promise for the future.

## CONFLICT OF INTEREST

K.L.H. is an employee of the U.S. Army. The views reported in this paper do not reflect the views of the Department of Defense or the United States Government. C.M.J. is an employee of the Maryland Veterans Affairs (VA) Health Care System at the Baltimore VA Medical Center. The views reported in this paper do not reflect the views of the Department of Veterans Affairs or the United States Government. C.M.J. holds an equity position in Cellth LLC.
